# Minimally Invasive Surgery through Right Mini-Thoracotomy for Mitral Valve Infective Endocarditis: Contraindicated or Safely Possible?

**DOI:** 10.3390/jcm13144182

**Published:** 2024-07-17

**Authors:** Maximilian Franz, Khalil Aburahma, Fabio Ius, Sadeq Ali-Hasan-Al-Saegh, Dietmar Boethig, Nora Hertel, Alina Zubarevich, Tim Kaufeld, Arjang Ruhparwar, Alexander Weymann, Jawad Salman

**Affiliations:** Department of Cardiothoracic, Transplantation and Vascular Surgery, Hannover Medical School, 30625 Hannover, Germanyweymann.alexander@mh-hannover.de (A.W.); salman.jawad@mh-hannover.de (J.S.)

**Keywords:** minimally invasive, mitral valve surgery, endocarditis, cardiac surgery

## Abstract

**Background**: Mitral valve infective endocarditis (IE) still has a high mortality. Minimally invasive mitral valve surgery (MIMVS) is technically more challenging, especially in patients with endocarditis. Here, we compare the early postoperative outcome of patients with endocarditis and other indications for MIMVS. **Methods**: Two groups were formed, one consisting of patients who underwent surgery because of mitral valve endocarditis (IE group: *n* = 75) and the other group consisting of patients who had another indication for MIMVS (non-IE group: *n* = 862). Patients were observed for 30 postoperative days. Data were retrospectively reviewed and collected from January 2011 to September 2023. **Results**: Patients from the IE group were younger (60 vs. 68 years; *p* < 0.001) and had a higher preoperative history of stroke (26% vs. 6%; *p* < 0.001) with neurological symptoms (26% vs. 9%; *p* < 0.001). No difference was seen in overall surgery time (211 vs. 206 min; *p* = 0.71), time on cardiopulmonary bypass (137 vs. 137 min; *p* = 0.42) and aortic clamping time (76 vs. 78 min; *p* = 0.42). Concerning postoperative data, the IE group had a higher requirement of erythrocyte transfusion (2 vs. 0; *p* = 0.041). But no difference was seen in the need for a mitral valve redo procedure, bleeding, postoperative stroke, cerebral bleeding, new-onset dialysis, overall intubation time, sepsis, pacemaker implantation, wound healing disorders and 30-day mortality. **Conclusions**: Minimally invasive mitral valve surgery in patients with mitral valve endocarditis is feasible and safe. Infective endocarditis should not be considered as a contraindication for MIMVS.

## 1. Introduction

Infective endocarditis can be considered a life-threatening infectious disease that affects the endocardium and heart valves. IE has an incidence of 3 to 10 per 100,000 patients per year, with a significant increasing trend [1]. Failure to promptly diagnose and manage IE can lead to high rates of morbidity and mortality. Complications associated with IE include heart failure, embolic events, abscess formation, sepsis, and septic shock [1,2,3].

The primary treatment for IE involves appropriate antibiotic therapy. When antibiotic therapy fails, mitral valve surgery plays a critical role in the management of infective endocarditis, particularly in cases with congestive heart failure, severe valvular regurgitation, persistent fever and bacteremia despite antibiotic therapy, systemic embolization, and large vegetations [1,4]. Over the years, significant advancements have been made in mitral valve surgery, with a growing emphasis on minimally invasive techniques with mini-thoracotomy approaches and progressing to robotic-assisted and video-assisted techniques [5,6].

In recent decades, the spectrum of indications for MIMVS has increasingly shifted and expanded to include more patients with a higher risk profile and more fragility and more complex mitral valve disease [4,5,6]. Careful patient selection is essential for the success of MIMVS [4,5,6]. Some surgeons consider endocarditis a contraindication for MIMVS based on several reasons, including, first, the limited access and reduced visibility associated with minimally invasive approaches, which may hinder the surgeon’s ability to adequately visualize and debride infected tissue. Second is the complexity of endocarditis cases, including the presence of large vegetations, abscesses, or valve destruction, which may require more extensive surgical intervention best achieved through traditional sternotomy [4,5,6].

The purpose of this study is to report our long-term experience in the treatment of infective mitral valve endocarditis using MIMVS with right mini-thoracotomy.

## 2. Materials and Methods

### 2.1. Study Population

Between January 2011 and September 2023, a total of 892 minimally invasive mitral valve surgeries were performed at our center. Among these, 75 surgeries were conducted on patients diagnosed with active infective endocarditis of the mitral valve (IE group). The non-IE group consisted of 862 patients who underwent minimally invasive mitral valve surgery for other surgical indications. Peri- and postoperative data were collected from our prospectively maintained database. In accordance with local German protocols, study approval by the institutional ethical review board was waived given the retrospective and non-interventional design of this study.

### 2.2. Surgical Technique

The surgical technique used in this study has been previously described by Salman et al. [7]. In summary, single lung ventilation was used, and cardiopulmonary bypass was generally established through the right inguinal vessels. Initially, a venous two-stage cannula was inserted into the superior vena cava under echocardiographic guidance, followed by the insertion of an arterial cannula. All surgeries were performed via a right mini-thoracotomy, with continuous insufflation of carbon dioxide throughout the procedure. The pericardium was opened 3–4 cm above the phrenic nerve. Unfractionated heparin was administered intravenously to achieve an activated clotting time above 450 s, and protamine was used for antagonization after venous cannula removal. In cases where weaning from cardiopulmonary bypass (CPB) resulted in right ventricular failure, veno-arterial extracorporeal membrane oxygenation (ECMO) was established. 

### 2.3. Follow-Up and Patient Data Collection

All patients were followed up for 30 days after surgery. Relevant clinical outcomes were recorded for all patients, including the incidence of postoperative ischemic stroke, right ventricular failure, new-onset atrial fibrillation, new-onset myocardial infarction, the need for pacemaker implantation, major bleeding requiring re-thoracotomy, sepsis, and renal failure requiring dialysis. Postoperative early mortality was defined as death occurring within hospital stay and the first 30 days after operation.

### 2.4. Echocardiographic Assessment

Baseline echocardiographic and hemodynamic evaluations were performed for all patients. Postoperative transthoracic echocardiography was routinely conducted prior to discharge to evaluate mitral valve function, with stenosis and insufficiency graded according to Omran et al. and Chew et al., respectively [8,9]. Pre- and postoperative echocardiographic features, including preoperative left ventricular ejection fraction (LVEF), rates of mitral valve insufficiency (MI) II, MI III, and MI IV, as well as rates of mitral valve stenosis (MS) II and MS III, were analyzed. The same features were also assessed postoperatively prior to hospital discharge.

### 2.5. Statistical Analysis

Data analysis was conducted using SPSS version 28.01.1. Continuous variables were described as the median and interquartile range (IQR). Categorical variables were presented as the number of cases and the percentage relative to the total group size. The Mann–Whitney U test was used to compare continuous variables, while the Chi-square test was employed for comparing categorical variables. Fisher’s exact test was performed when at least one cell of the crosstab had an expected count less than 5. A *p*-value of less than 0.05 was considered statistically significant.

## 3. Results

### 3.1. Patient Characteristics

Patients in the IE group were younger compared to those in the non-IE group. They had a higher incidence of preoperative stroke and more frequent occurrence of neurological symptoms. In contrast, the IE group had a lower rate of preoperative pulmonary hypertension, coronary artery disease, arterial hypertension, hyperlipidemia, and atrial fibrillation. Mitral valve stenosis (0% vs. 7%; *p =* 0.011), severe mitral valve regurgitation (5% vs. 94%; *p* < 0.001) and annulus dilatation (23% vs. 50%; *p =* 0.001) were more prevalent in comparison with the non-IE group (Table 1).

### 3.2. Procedural Outcomes 

There was no considerable difference in operation time, cardiopulmonary bypass time or aortic cross-clamp time between the groups. Patients in the IE group more often received mechanical valve replacement (24% vs. 10%; *p =* 0.002), while patients in the non-IE group more often underwent mitral valve repair (44% vs. 64%; *p =* 0.001) and concomitant tricuspid procedures (5% vs. 14%; *p =* 0.033) (Table 2).

Postoperatively, there were no significant differences between the IE and non-IE groups in the incidence of stroke (3% vs. 2%; *p =* 0.65), intracerebral bleeding (1% vs. 0%; *p =* 0.29), delirium (4% vs. 3%; *p =* 0.72), sepsis (1% vs. 2%; *p =* 1), or new-onset myocardial infarction (0% vs. 0%; *p =* 1). The groups also did not differ in the rates of renal insufficiency with new-onset dialysis (5% vs. 3%; *p =* 0.29), right ventricular failure requiring ECMO (3% vs. 3%; *p =* 1), re-thoracotomy for bleeding (12% vs. 7%; *p =* 0.11), in-hospital mortality (4% vs. 2%; *p =* 0.084) or 30-day mortality (5% vs. 2%; *p =* 0.24). However, patients in the non-IE group had a higher postoperative incidence of new-onset atrial fibrillation (4% vs. 10%; *p =* 0.002) and arrhythmia in general (3% vs. 13%; *p =* 0.005), although there was no difference in pacemaker implantation rate (1% vs. 6%; *p =* 0.12) between the two groups (Table 3).

### 3.3. Echocardiographic Results

Postoperatively, there were no differences between the groups in ejection fraction (55% vs. 53%; *p =* 0.74), mitral valve insufficiency grade 1 (9% vs. 19%; *p =* 0.1), mitral valve insufficiency grade 2 (1% vs. 2%; *p =* 1), or mitral valve stenosis grade 2 (0% vs. 2%; *p =* 1). Mitral valve insufficiency grade 3 or 4, as well as mitral valve stenosis grade 3, were not observed in either group (Table 4).

## 4. Discussion

Over the past few years, there has been continuous development in the field of MIMVS. Experienced heart valve centers now routinely perform operations using a right mini-thoracotomy approach for complex valve diseases and high-risk patient subgroups, including those with infective endocarditis [5,10,11]. Despite the positive outcomes reported in the literature and the recognized advantages, the adoption of minimally invasive surgery for IE remains limited. Concerns have been raised regarding the challenges associated with the surgical learning curve, which could potentially compromise the effectiveness of the procedure and the ability to achieve optimal valve repair, particularly in complex valve diseases [11,12,13].

Our single-center retrospective analysis showed that patients from the IE group had a higher preoperative incidence of stroke and neurological symptoms, and mechanical valve prothesis was more often used in comparison to the non-IE group. However, the higher proportion of neurological disorders preoperatively was not reflected in the postoperative outcomes where the stroke rate in IE group was not more significant than in the non-IE group. Furthermore, the incidence of other postoperative complications including sepsis, myocardial infarction, right ventricular failure and renal insufficiency with new-onset dialysis in the patients of the IE group was low like in the non-IE group.

In a recent study by Barbero et al., positive early and long-term outcomes were reported in higher-risk patients who underwent minimally invasive surgery for mitral valve infective endocarditis [11]. The authors highlighted the crucial role of comprehensive screening, including total body, vascular and echocardiographic assessments, in selecting the most suitable approach [11]. This screening process enables the extension of indications for minimally invasive surgery to include patients with more severe conditions, such as active endocarditis and sepsis [11].

Folkmann et al. performed a retrospective, single-center analysis with a follow-up period of one year which analyzed the outcome of 92 patients who underwent MIMVS for mitral valve endocarditis [14]. They reported a mitral valve repair rate of 24%, postoperative stroke rate of 4%, sepsis rate of 2%, ECMO rate of 1% and dialysis rate of 13%. The 30-day mortality was 9.8% [14]. These results are slightly different from our results. We report a mitral valve repair rate of 44%, 30-day mortality of 5% and stroke, sepsis, ECMO and dialysis rates of 3%, 1%, 3% and 5%, respectively. To sum up, the complication rates in our study and in the study of Folkmann et al. are remarkably low, which confirms the safety and effectivity of MIMVS for mitral valve endocarditis.

Kofler et al. reported a shorter overall operation time, less blood transfusion and shorter ventilation time and finally concluded that the minimally invasive approach is superior to sternotomy in selected patients [15]. Their overall surgery time was shorter compared to the overall surgery time in the patients of our study, while their ventilation time was comparable to the ventilation time of the patients in our study group. The 30-day mortality was also identical.

Van Praet et al. stated in a case report that MIMVS for endocarditis reduces the risk of wound infections and consequently the risk of redo procedures [16]. We can confirm this statement through our results which show a wound infection rate of 5%. Van Praet et al., furthermore, stated that mitral valve replacement is acceptable if the endocarditis does not involve the anulus [16]. This is also in agreement with our work which has a replacement rate of 56% with a low rate of recurrence.

According to our findings and experience, MIMVS can hold great promise as an effective and less invasive treatment option for IE. This approach offers several important benefits, including reduced surgical trauma and better recovery with improved outcomes. To sum up, there is a limited amount of literature describing the short- and long-term outcomes after MIMVS in mitral valve endocarditis; however, the common statement of the available literature is clear: infective endocarditis should not be considered a contraindication for MIMVS, and the outcomes are clinically acceptable. Our work underlines this important statement.

## 5. Conclusions

Minimally invasive mitral valve surgery through right mini-thoracotomy for infective mitral valve endocarditis had no negative impact on the early postoperative outcome. Therefore, endocarditis should not be seen as a contraindication for minimally invasive mitral valve surgery. This study has several limitations that should be considered when interpreting the results. Firstly, the analysis was not conducted using propensity score matching. Additionally, the data only represented the early follow-up period, which could influence the results. To address these limitations, further research is needed to comprehensively compare the mid-term and long-term clinical outcomes of MIMVS in patients with IE versus non-IE.

## Figures and Tables

**Table 1:** Preoperative data.

Variables	IE (*n* = 75)	non-IE (*n* = 862)	*p*-Value
Preoperative characteristics			
Age	60 (50–69)	68 (58–75)	<0.001
Female gender	26 (34%)	400 (46%)	0.054
Cardiac reoperation	11 (15%)	96 (11%)	0.35
Chronic kidney disease	16 (21%)	148 (17%)	0.43
Hemodialysis	5 (7%)	35 (4%)	0.24
Extracardiac arteriopathy	2 (2.7%)	67 (8%)	0.16
Chronic obstructive lung disease	7 (9%)	133 (15%)	0.18
Recent pneumonia	0 (0%)	1 (0%)	1
Insulin dependent diabetes mellitus	2 (2.7%)	33 (4%)	0.84
Recent myocardial infarction	1 (1%)	12 (1%)	1
History of stroke	20 (27%)	54 (6%)	<0.001
Neurologic symptoms	20 (27%)	77 (9%)	<0.001
Pulmonary hypertension	14 (19%)	399 (46%)	<0.001
Coronary artery disease	11 (15%)	250 (29%)	0.01
Smoking history	13 (17%)	190 (22%)	0.38
Arterial hypertension	37 (49%)	591 (69%)	0.001
Hyperlipidemia	22 (29%)	401 (47%)	0.004
Atrial fibrillation	19 (25%)	405 (47%)	<0.001
Operative indications			
Mitral valve stenosis	0 (0%)	57 (7%)	0.011
Mitral valve regurgitation	4 (5%)	809 (94%)	<0.001
Mitral valve prolapse	33 (44%)	513 (60%)	0.01
Mitral chord rupture	26 (35%)	355 (41%)	0.27
Anulus dilatation	17 (23%)	435 (50%)	0.001
Calcified anulus	0 (0%)	14 (2%)	1

**Table 2:** Intraoperative data.

Variables	IE (*n* = 75)	Non-IE (*n* = 862)	*p*-Value
Surgery time (minutes)	211 (172–251)	205 (176–239)	0.64
Time on CPB (minutes)	137 (114–180)	136 (113–164)	0.40
Aortic cross-clamp time	76 (61–104)	77 (59–95)	0.49
Mitral valve replacement			
Biological	24 (32%)	222 (26%)	0.27
Mechanical	18 (24%)	90 (10%)	0.002
Mitral valve repair	33 (44%)	550 (64%)	0.001
Concomitant tricuspid procedure	4 (5%)	122 (14%)	0.033

**Table 3:** Postoperative data.

Variables	IE (*n* = 75)	Non-IE (*n* = 862)	*p*-Value
Catecholamine duration	17 (6–39)	17 (9–27)	0.98
Ventilation time (hours)	11 (8–20)	11 (7–16)	0.12
ICU stay (days)	1 (0–2)	1 (0–2)	0.91
Mitral valve reoperation	3 (4%)	10 (1%)	0.81
Arrhythmia	2 (3%)	110 (13%)	0.005
Pneumothorax	2 (3%)	46 (5%)	0.42
ECMO/right ventricular failure	2 (3%)	29 (3%)	1
Re-thoracotomy/bleeding	9 (12%)	60 (7%)	0.11
New atrial fibrillation	3 (4%)	87 (10%)	0.002
Wound healing disorder	4 (5%)	57 (7%)	0.81
Renal insufficiency with new-onset dialysis	4 (5%)	26 (30%)	0.29
Stroke	2 (3%)	16 (2%)	0.65
Delirium	3 (4%)	26 (3%)	0.72
Cerebral bleeding	1 (1%)	3 (0.3%)	0.29
Periph. vascular complications	1 (1%)	4 (0.4%)	0.35
Sepsis	1 (1%)	14 (2%)	1
Myocardial infarction	0 (0%)	2 (0.2%)	1
Pacemaker implantation	1 (1%)	52 (6%)	0.12
30-day mortality	4 (5%)	18 (2%)	0.24
Intrahospital mortality	3 (4%)	17 (2%)	0.084

**Table 4:** Echocardiographic measurements.

Time	Parameter	IE (*n* = 75)	Non-IE (*n* = 862)	*p*-Value
Preoperative	LVEF	60 (55–65)	60 (51–68)	0.012
	MI III	30 (40%)	568 (66%)	0.001
	MI IV	6 (8%)	77 (9%)	1
	MS II	0 (0%)	15 (2%)	0.62
	MS III	0 (0%)	26	0.03
Postoperative	LVEF	55 (45–60)	53 (45–60)	0.74
	MI I	7 (9%)	163 (19%)	0.1
	MI II	1 (1%)	20 (2%)	1
	MI III	0 (0%)	0 (0%)	-
	MI IV	0 (0%)	0 (0%)	-
	MS II	0 (0%)	20 (2%)	1
	MS III	0 (0%)	0 (0%)	-

## Data Availability

The original contributions presented in the study are included in the article, further inquiries can be directed to the corresponding author.
